# Research Trends of Patient-Reported Outcome Measures in Orthopedic Medical Practices: A Bibliometric and Visualized Study

**DOI:** 10.3390/medicina59091664

**Published:** 2023-09-14

**Authors:** Hongfu Jin, Miao He, Wenqing Xie, Zixuan Xiong, Zhenhan Deng, Yusheng Li

**Affiliations:** 1 Department of Orthopedics, Xiangya Hospital, Central South University, Changsha 410017, China; 2 National Clinical Research Center for Geriatric Disorders, Xiangya Hospital, Central South University, Changsha 410017, China; 3 Xiangya School of Medicine, Central South University, Changsha 410017, China; 4 Department of Sports Medicine, The First Affiliated Hospital of Shenzhen University, Shenzhen Second People’s Hospital, Shenzhen 518037, China

**Keywords:** patients, PROMs, orthopedic, function, publication, bibliometric analysis, web of science

## Abstract

*Background and Objectives*: Patient-reported outcome measures (PROMs), also known as self-report measures, are critical tools for evaluating health outcomes by gathering information directly from patients without external interpretation. There has been a growing trend in the number of publications focusing on PROMs in orthopedic-related research. This study aims to identify the most valuable publications, influential journals, leading researchers, and core countries in this field using bibliometric analysis, providing researchers with an understanding of the current state and future trends of PROMs in orthopedic research. *Materials and Methods*: All PROMs in orthopedic-related publications from 1991 to 2022 were obtained from the WoSCC database. R software (version 4.2.2), VOSviewer (version 1.6.17), and Microsoft Excel (version 2303) were used for the bibliometric and visual analysis. *Results*: A total of 2273 publication records were found from 1991 to 2022. The results indicated that the United States (US) has made significant contributions to orthopedic-related PROMs. The majority of active research institutions are located in the US. *J ORTHOP RES* has published the most articles. *J BONE JOINT SURG AM* has the highest total citations. *Conclusions*: Our study provides a valuable reference for further exploration of the application of PROMs in orthopedics. PROMs have emerged as an increasingly popular area of research within the field of orthopedics, both in clinical practice and academic research. We conducted a bibliometric analysis in terms of journals, authors, countries, and institutions in this field. Additionally, we analyzed the potentialities and advantages of using PROMs in orthopedic research. There is an increasing trend towards using network-based or short message service (SMS)-based electronic patient-reported outcome measures (ePROMs) in orthopedic medical practices. It is anticipated that the role of PROMs in psychological and mental health research and telemedicine will continue to grow in importance.

## 1. Introduction

Patient-centered outcomes research (PCOR) and patient-centered care (PCC) have received increasing attention in medical practices over the past few decades, indicating that patient factors have now been widely recognized as a critical aspect of medical practices by healthcare providers worldwide [1]. Patient-reported outcome measures (PROMs), also called self-report measures, have been developed and used since the 1970s to gather information about patients’ health status and their perceptions of health-related quality of life (QoL), which allows patients to report their experiences with illnesses, symptoms, and treatment outcomes in a standardized way [2,3]. PROMs are essential tools for assessing health outcomes by collecting information directly from patients without external interpretation, which offers valuable insights into the impact of diseases and interventions on patients’ health [4]. PROMs encompass a broad spectrum of aspects pertaining to patient healthcare. These include: (1) reports on patients’ health status and health-related QoL; (2) reports on patients’ health behaviors (both harmful and beneficial); (3) reports on patients’ satisfaction; (4) reports on patients’ well-being; and (5) reports on patients’ symptoms and functioning [3,5]. PROMs can be divided into generic and disease- or condition-specific measures. Generic PROMs are not tailored to a specific population and thus can be applied to a wide range of patient groups and the general population but may not be as relevant to the respondent or sensitive to detecting changes in the patient’s health status [6]. Additionally, generic PROMs may include content unrelated to the patient’s condition, leading to patients feeling neglected or ignored or refusing to answer questions they deem irrelevant. Disease- or condition-specific PROMs are designed for patient groups or a unique clinical problem and can be used to measure and collect data specific to the patient’s condition [7,8].

PROMs have become a fundamental component of both academic and clinical research. These measures offer valuable insights into patient pain, functionality, and QoL during the perioperative period of orthopedic surgery [9,10,11]. By gathering data directly from patients, PROMs provide a unique perspective on the experiences of patients. Orthopedic injuries that involve extensive damage to tissues, bones, and blood vessels can require a lengthy recovery process. Patients may experience pain, movement limitations, and decreased function [12]. Orthopedic surgery can pose challenges for patients with orthopedic injuries in terms of managing postoperative pain and physical limitations [13,14]. Nevertheless, it is crucial to prioritize functional recovery and QoL for patients undergoing such procedures. Ensuring that patients can restore their physical capabilities and maintain a high level of well-being following the procedure is of utmost importance. PROMs can play a vital role in this process by providing a means for patients to convey their experiences and needs to their healthcare providers. As effective instruments for both patients and clinicians, PROMs can facilitate communication and improve patient outcomes [15,16].

Bibliometric analysis is a quantitative analysis of large-scale academic publications that is used to provide insights into research hotspots, future trends, and the academic impact of a particular field. In the field of orthopedic-related research, there has been a steady increase in publications focused on PROMs. The purpose of this study is to conduct a bibliometric analysis to help identify the most valuable publications, influential journals, leading researchers, and core countries in this field, providing researchers with an understanding of the current state and future trends of PROMs in orthopedic-related research.

## 2. Materials and Methods

### 2.1. Data Source

The Web of Science Core Collection (WoSCC) database is a widely used literature retrieval database that comprises high-quality academic literature covering a vast range of fields of study. The original data retrieved from the WoSCC database include references cited in publications, which are indexed based on the authors, sources, and publication date. Compared to other databases, the WoSCC database is more accurate in classifying publication types. With all these distinctive features, the WoSCC database caters to the bibliometric analysis needs of PROMs in orthopedic-related research in our study.

### 2.2. Search Strategy

**Figure 1:**
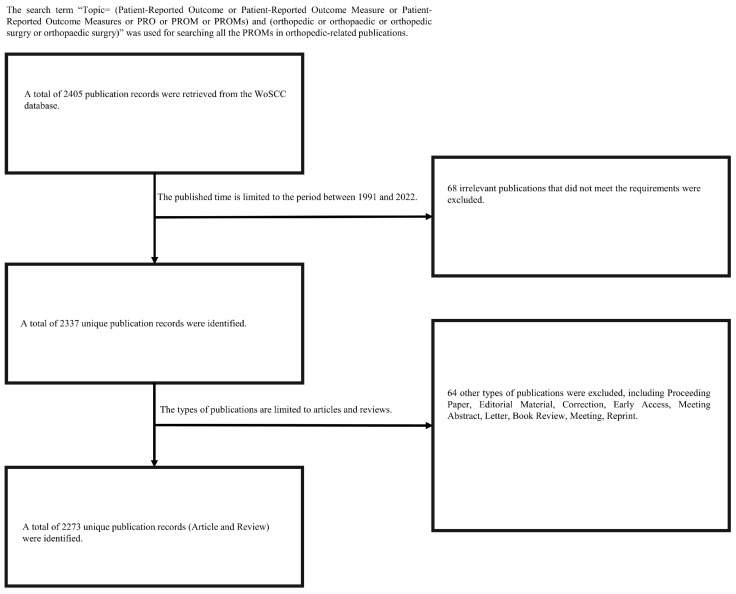
Flowchart of the search strategy for our bibliometric and visualized analysis.

Our bibliometric analysis was conducted on 14 April 2023. The following search strategy was used to identify all publications related to PROMs in orthopedic research in the WoSCC database: “Topic = (Patient-Reported Outcome or Patient-Reported Outcome Measure or Patient-Reported Outcome Measures or PRO or PROM or PROMs) and (orthopedic or orthopaedic or orthopedic surgery or orthopaedic surgery)”. The publication time was set between 1991 and 2022 and was restricted to articles and reviews. The retrieved publication records were exported in “plain text” format, with “full records and cited references”, and these exported data were used as the raw data for our bibliometric analysis. The search strategy flowchart for this study is presented in Figure 1.

### 2.3. Data Analysis Tools

In our study, we employed several tools to conduct a comprehensive bibliometric analysis. The open-source “bibliometrix” package, based on the R language platform (version 4.2.2) [17,18], provided a range of functions for analyzing and visualizing bibliometric data, allowing us to assess the impact and contributions of authors, journals, and institutions. VOSviewer (version 1.6.17) was used to perform various types of bibliometric analysis, including co-occurrence analysis, co-citation analysis, and bibliographic coupling analysis, revealing connections and relationships among publications. Finally, we used Microsoft Excel (version 2303) to generate bar charts and line graphs to aid in the visualization of PROMs in orthopedic-related research.

## 3. Results

### 3.1. Overview of Included Publications

**Figure 2:**
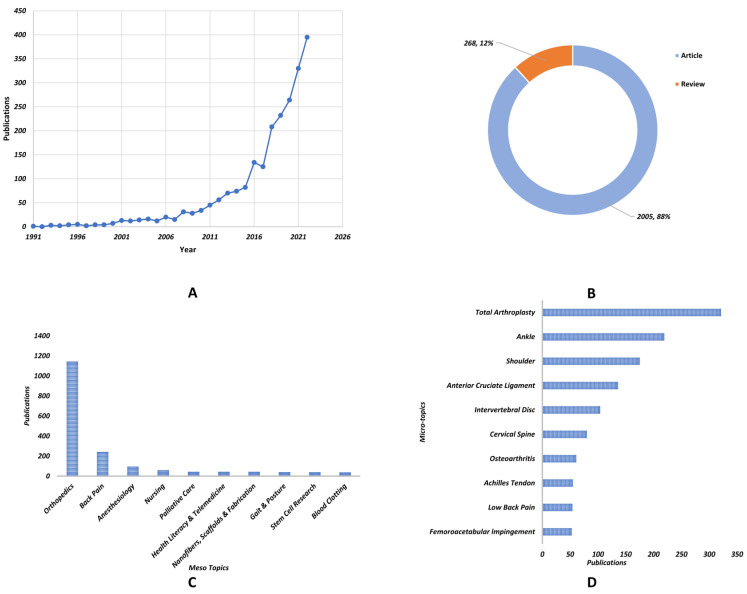
**Overview of Included Publications.** (**A**) The annual distribution of publications from 1991–2022. (**B**) Types of publications. (**C**) Meso-topics of included publications. (**D**) Micro-topics of the included publications.

A total of 2273 publication records were included in our study. Figure 2A illustrates the annual distribution of publications, revealing several distinct phases in the evolution of research trends. During the initial phase, spanning from 1991 to 2000, the volume of publications remained consistently low, with only a handful of articles published each year. The subsequent phase, from 2001 to 2015, was characterized by a gradual yet persistent increase in publication volume. The final phase, from 2016 to 2022, witnessed a rapid surge in the number of publications. Despite a minor dip in publication volume in 2017, it quickly rebounded in 2018. The observed trend suggests a growing interest in PROM research in the field of orthopedics. Figure 2B indicates that the majority of publications within this field are articles, comprising approximately 88% of the total. Figure 2C outlines the meso topics of publications within this domain, with orthopedics representing the largest proportion of publications, followed by back pain, anesthesiology, nursing, and palliative care. Figure 2D presents the micro topics of publications within this field, with total arthroplasty being the most prevalent topic, followed by ankle, shoulder, anterior cruciate ligament, and intervertebral disc.

### 3.2. Journal Analysis

**Figure 3:**
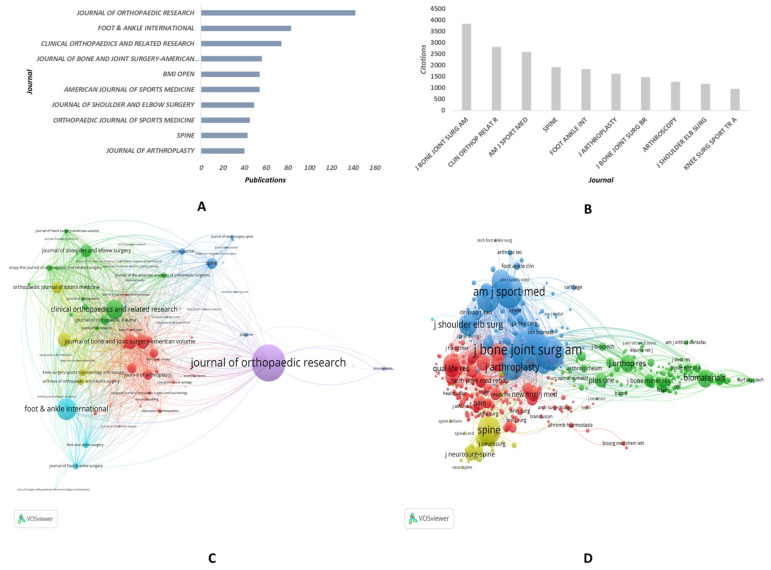
**Journal analysis of PROMs in orthopedic-related publications from 1991–2022.** (**A**) The number of publications in the top 10 most relevant journals. (**B**) The total citations of the top 10 most highly cited journals. (**C**) The bibliographic coupling analysis of the relevant journals. (**D**) Co-citation analysis of the relevant journals.

In terms of the number of publications, *JOURNAL OF ORTHOPAEDIC RESEARCH* ranks first, with a total of 142 publications published between 1991 and 2022, followed by *FOOT & ANKLE INTERNATIONAL* (83 publications), *CLINICAL ORTHOPAEDICS AND RELATED RESEARCH* (74 publications), *JOURNAL OF BONE AND JOINT SURGERY-AMERICAN VOLUME* (56 publications), and *BMJ OPEN* (54 publications) (Figure 3A). In terms of total citations, *J BONE JOINT SURG AM* ranks first with a cumulative total of 3838 citations, followed by *CLIN ORTHOP RELAT R* (2818 citations), *AM J SPORT MED* (2602 citations), *SPINE* (1925 citations), and *FOOT ANKLE INT* (1831 citations) (Figure 3B). VOSviewer was used to generate visualizations of the bibliographic coupling and co-citation analysis of the journals. Each circle in the bibliographic coupling analysis represents a journal, with its size denoting the number of publications. The distance between circles indicates the strength of the bibliographic coupling relationship, with closer distances signifying stronger relationships. Clusters of closely positioned circles represented groups of frequently bibliographically coupled journals. As evident in Figure 3C, 93 journals met the minimum threshold of five documents for the bibliographic coupling analysis, resulting in the classification of all journals into six clusters, with the largest cluster comprising 30 journals. Similarly, in the co-citation analysis, each circle represented a journal, and its size denoted the frequency of citation. The proximity between circles represents the strength of the co-citation relationship between the two journals, with closer circles indicating stronger associations. The co-citation analysis classified all journals into six clusters, with the largest cluster comprising 214 journals (Figure 3D).

### 3.3. Country and Institution Analysis

**Figure 4:**
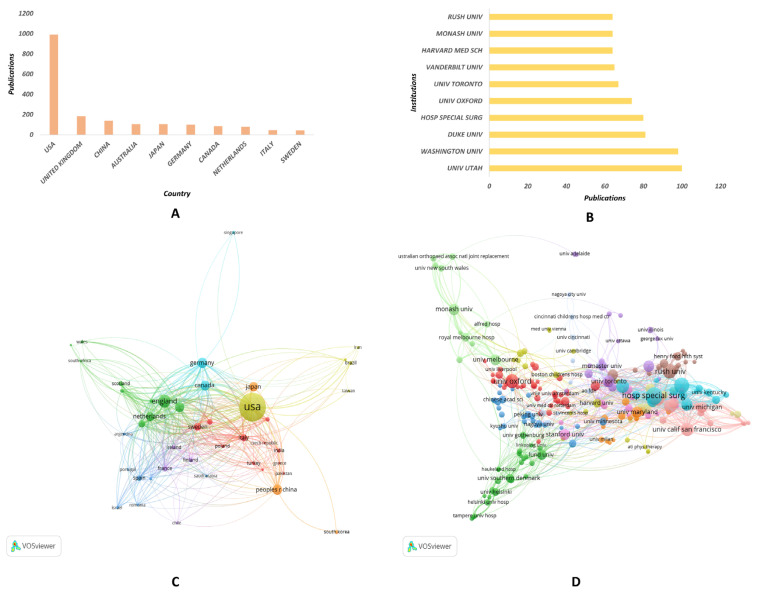
**Country and Institution Analysis of PROMs in orthopedic-related publications from 1991–2022.** (**A**) The number of publications of the top 10 most relevant countries. (**B**) The total citations of the top 10 most highly cited journals. (**C**) The bibliographic coupling analysis of the relevant journals. (**D**) Co-citation analysis of the relevant journals.

The United States has published the most publications related to PROMs in orthopedic research, with a total of 933 publications. The United Kingdom ranks second, with 183 publication records. In addition, PROMs in orthopedic research have also attracted attention in China, Australia, Japan, and other countries (Figure 4A). Among the global research institutions that are highly regarded in the field of PROMs in orthopedic research, the top five institutions with the most publications are all located in the US, including UNIV UTAH (100 publications), WASHINGTON UNIV (98 publications), DUKE UNIV (81 publications), HOSP SPECIAL SURG (80 publications), and UNIV OXFORD (74 publications) (Figure 4B). In the VOSviewer visualization of the co-authorship analysis of country or institution, each country or institution is represented by a circle, and the size of the circle reflects the number of publications with authors from that country or institution. The distance between two circles represents the strength of the co-authorship relationship between the two countries or institutions, with closer circles indicating a stronger relationship. Clusters of closely positioned circles represent groups of countries or institutions that frequently collaborate on publications. Figure 4C shows that countries such as the US, UNITED KINGDOM, and CHINA have established extensive cooperation relationships globally. Figure 4D shows that research institutions such as UNIV UTAH, WASHINGTON UNIV, DUKE UNIV, HOSP SPECIAL SURG, and UNIV OXFORD have established extensive cooperation relationships globally in this field.

### 3.4. Author Analysis

**Figure 5:**
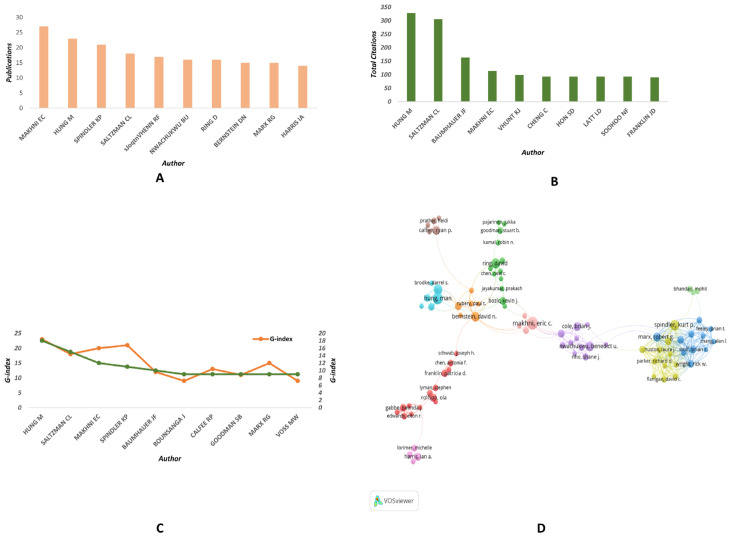
**Author Analysis of PROMs in Orthopedic-Related Publications from 1991–2022.** (**A**) The number of publications by the top 10 most relevant authors. (**B**) The total citations of the top 10 most highly cited authors. (**C**) The H-index and G-index of the top 10 most highly cited authors. (**D**) Co-authorship analysis of relevant authors.

In terms of the number of publications, MAKHNI EC emerges as the most productive author within this field. Additionally, HUNG M, SPINDLER KP, SALTZMAN CL, and VHENN RF also demonstrated considerable activity in this field (Figure 5A). In terms of total citations, HUNG M ranks as the preeminent author in this field (Figure 5B). The H-index and G-index represent two widely used metrics for assessing the research impact of authors. As evidenced by Figure 5C, Hung Man possesses the highest H-index and G-index and can thus be regarded as the most influential author within this field. The co-authorship analysis network depicted in Figure 5D reveals that Hung Man has forged close collaborative relationships with researchers such as Saltzman Charles L., Voss Maren W., and Bounsanga Jerry within the field of PROMs in orthopedic research.

### 3.5. Keyword Analysis

**Figure 6:**
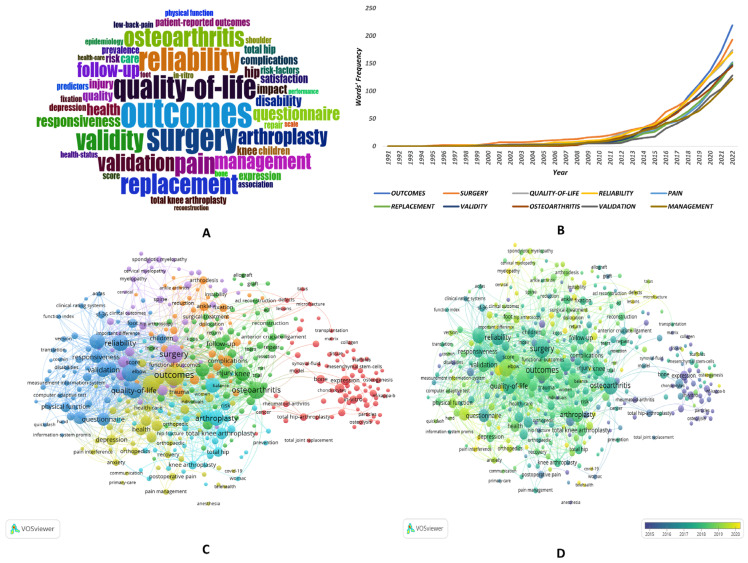
**Keyword Analysis of PROMs in Orthopedic-Related Publications from 1991–2022.** (**A**) The word cloud of keywords. (**B**) The frequency of the top 10 high-frequency keywords over time. (**C**) The keyword co-occurrence network. (**D**) Overlay visualization of the keyword co-occurrence network.

Figure 6A presents a word cloud of keywords in the field, where the size of each word indicates its frequency of occurrence. The keyword “outcomes” had the highest frequency at 223 occurrences, followed by “surgery” (197 occurrences), “quality-of-life” (177 occurrences), “reliability” (174 occurrences), and “pain” (155 occurrences). Figure 6B illustrates that the top 10 high-frequency keywords in the field have experienced an upward trend in popularity over time. The keyword co-occurrence network, generated by VOSviewer and displayed in Figure 6C,D, comprises a total of 392 keywords that meet the minimum occurrence threshold of 10. The resulting visualization displays clusters of related keywords, with the size of each keyword reflecting its frequency. All these keywords are categorized into seven clusters, with larger clusters suggesting that “The translation and validation of PROM Tools in orthopedics”, “PROMs in Arthroplasty”, and “PROMs in sports medicine” are popular research topics. Figure 6D highlights the emergence of keywords such as “Telehealth”, “Telemedicine”, and “Mental Health”, indicating potential future research trends in the field.

## 4. Discussion

### 4.1. Current Research on PROMs in Orthopedic Research

Bibliometric analysis is a valuable tool for characterizing and analyzing the evolving landscape of a particular research field [19]. In our study, a bibliometric analysis was conducted to analyze PROMs in orthopedic-related publications published between 1991 and 2022. Over the years, the role of PROMs in orthopedic clinical and academic research has attracted increasing attention. A large number of publications on this topic have been published. Based on publication data retrieved from the WoSCC database, we analyzed basic information about these publications, including annual publications, the most productive and influential authors, countries/regions, institutions, journals, and keywords. In addition, we evaluated the current state and hotspots of research on PROMs in orthopedic clinical and academic research. The United States has the highest number of publications, and the top five related institutions are all located in the United States, indicating that the United States is the most active country in this field of research.

The trend of annual output serves as an indicator of the development process of PROMs in orthopedic research, which presents an increasing trend over time. This suggests that research on PROMs in orthopedics is likely to remain a prominent research focus in the future. In terms of journals, *JOURNAL OF ORTHOPAEDIC RESEARCH* published the most relevant publications. Compared with other journals, *J BONE JOINT SURG AM* has the most citations. Future researchers should pay more attention to this journal to understand high-quality research in this field. The US has the highest number of publications, and the top five related institutions are all located in the US, indicating that the US is the most active country in this field of research. UNIV UTAH published the most publications among all institutions, making it an active participant in this field. Widespread collaboration among various countries and institutions is observed. Professor MAKHNI EC is the most productive author, while HUNG M has the most citations. Judging from the H-index and G-index, Professor HUNG M has the most influence in this field, indicating that his publications are of a high quality and are worth studying. The keyword analysis showed that the current application of PROMs in arthroplasty and sports medicine is relatively widespread.

### 4.2. Potentialities and Advantages of PROMs in Orthopedic Research

**Figure 7:**
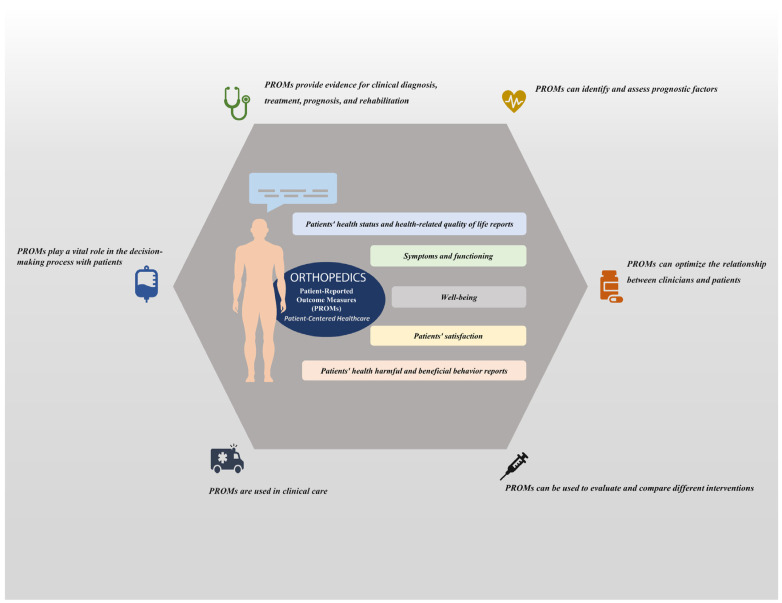
Potentialities and advantages of PROMs in orthopedic-related research.

PROMs have emerged as a valuable source of evidence for clinical diagnosis, treatment, prognosis, and rehabilitation in orthopedic-related research. Therefore, the proper implementation of PROMs in orthopedic medical practices holds significant value for both academic and clinical research [20]. PROMs can facilitate patient involvement in the decision-making process, improve compliance, and aid in the selection of appropriate interventions. Effective communication and trust between patients and clinicians are essential for shared decision-making (SDM), which ultimately leads to improved patient satisfaction [21,22]. Moreover, PROMs can be used to assess the quality of clinical healthcare from the patient’s perspective and help improve the quality of healthcare. There is a growing international focus on using PROMs to provide patient-centered care and increase patient engagement and satisfaction in orthopedic clinical practices [23]. By directly involving patients in assessing and reporting their own outcomes, PROMs enable them to actively contribute their unique insights and experiences to the clinical decision-making process. Additionally, PROMs can identify and assess the prognostic factors of patients undergoing orthopedic surgery, which is essential for evaluating prognostic factors and developing recovery and rehabilitation strategies [24,25,26]. Perioperative evaluations of patients using PROMs can provide a comprehensive understanding of their expectations and enhance the relationship between clinicians and patients. Patient satisfaction after the orthopedic procedure is strongly associated with postoperative outcomes during the follow-up period [27,28]. Therefore, PROMs are an important tool for assessing the safety, efficacy, and cost-effectiveness of various interventions, as well as improving patient-centered clinical care and patient satisfaction (Figure 7).

### 4.3. Future Perspectives of PROMs in Orthopedic Research

PROMs are reliable and valid tools for quantifying patients’ perspectives on the impact of diseases and interventions on their health and are widely used in clinical healthcare and orthopedic-related academic and clinical research. PROMs are considered an integral component of COS, and data on PROMs can be obtained by searching databases constructed by professional organizations such as the Core Outcome Measures in Effectiveness Trials (COMET) and the Outcome Measures in Rheumatology [29,30]. There is a growing interest in utilizing core outcome sets (COS) to measure and report specific health conditions in clinical trials [31]. The credibility of PROM data collection and analysis must be ensured by using scientifically rigorous tools with high reliability, validity, and sensitivity to detect changes in patients. Furthermore, it is crucial to develop questionnaires that assess the physical and psychosocial aspects of specific populations while considering language and cultural differences that may affect the accuracy of the data collected [32,33]. Therefore, it is important to select the appropriate PROM tools by referring to instruments used in previous studies or considering the application scope of the tools. The application of computerized adaptive testing (CAT) can potentially reduce the burden on patients and clinicians while maintaining the reliability and validity of outcomes [34,35]. Moreover, there is an increasing trend towards using electronic patient-reported outcome measures (ePROMs) that are network-based and short message service (SMS)-based, which could increase response rates, reduce burden, and collect valuable information from patients. Evidence suggests that patients are willing to complete ePROM symptom questionnaires. Moreover, the collected data can be integrated into electronic patient records (EPR) [36,37]. As telemedicine continues to gain popularity in orthopedic medical practices, the importance of PROMs in orthopedic telemedicine may garner increased attention. By providing doctors with valuable insights into patients’ symptoms and overall health status, PROMs have the potential to enhance the quality of telemedicine services for patients in the future. Furthermore, the analysis of keywords indicates that the role of PROMs in psychological and mental health research has garnered increased concerns within the orthopedic field, particularly with regard to depression [38,39].

### 4.4. Limitations

Some limitations need to be clarified. First, selecting the WoSCC database as a single source may inevitably result in an incomplete data analysis. Some relevant publications may be excluded from the analysis, leading to potential bias or an incomplete representation of the research landscape. Second, due to the continuous updating of data in the WoSCC database, some recently published publications may be ignored and cause the results to lag behind actual research progress, which is one of the common limitations inherent in bibliometric studies. Third, citation is frequently used as a metric to assess the influence and interconnectedness of research in bibliometric analysis. Some significant research findings may not receive widespread citations, while low-quality publications may receive a higher number of citations, which can affect the accuracy and objectivity of the analysis results.

Nonetheless, an exclusive reliance on citation relationships may introduce a bias in the form of citation bias. This bias can manifest as the underrepresentation of noteworthy research findings in terms of citation count, while comparatively insignificant or substandard publications may garner a disproportionately higher number of citations. Consequently, the presence of citation bias can compromise the precision and impartiality of the analysis outcomes.

## 5. Conclusions

Our study provides a valuable reference for further exploration of the application of PROMs in orthopedics. PROMs have emerged as an increasingly popular area of research within the field of orthopedics, both in clinical practice and academic research. The US can be considered the leader in this area of research, with the University of Utah being recognized as the most relevant institution that has established a broad network of global collaborations. *JOURNAL OF ORTHOPAEDIC RESEARCH* has published the most related publications, while *J BONE JOINT SURG AM* is the journal with the most citations in this field. Additionally, we analyzed the potentialities and advantages of using PROMs in orthopedic research. There is an increasing trend towards using network-based or short message service (SMS)-based electronic patient-reported outcome measures (ePROMs) in orthopedic medical practices. It is anticipated that the role of PROMs in psychological and mental health research and telemedicine will continue to grow in importance.

## Data Availability

The datasets used and/or analyzed during the research are available from the corresponding author upon reasonable request.
